# Genetic diversity of vaccine candidate antigens in *Plasmodium falciparum *isolates from the Amazon basin of Peru

**DOI:** 10.1186/1475-2875-7-93

**Published:** 2008-05-27

**Authors:** Stella M Chenet, OraLee H Branch, Ananias A Escalante, Carmen M Lucas, David J Bacon

**Affiliations:** 1 Parasitology Program, Naval Medical Research Center Detachment, Lima, Peru; 2 Department of Medicine, University of Alabama at Birmingham, Birmingham, AL, USA; 3 Arizona State University, School of Life Sciences, Tempe, AZ, USA; 4 Navy Environmental and Preventive Medicine Unit 2, Norfolk, VA, USA

## Abstract

**Background:**

Several of the intended *Plasmodium falciparum *vaccine candidate antigens are highly polymorphic and could render a vaccine ineffective if their antigenic sites were not represented in the vaccine. In this study, characterization of genetic variability was performed in major B and T-cell epitopes within vaccine candidate antigens in isolates of *P. falciparum *from Peru.

**Methods:**

DNA sequencing analysis was completed on 139 isolates of *P. falciparum *collected from endemic areas of the Amazon basin in Loreto, Peru from years 1998 to 2006. Genetic diversity was determined in immunological important regions in circumsporozoite protein (CSP), merozoite surface protein-1 (MSP-1), apical membrane antigen-1 (AMA-1), liver stage antigen-1 (LSA-1) and thrombospondin-related anonymous protein (TRAP). Alleles identified by DNA sequencing were aligned with the vaccine strain 3D7 and DNA polymorphism analysis and FST study-year pairwise comparisons were done using the DnaSP software. Multilocus analysis (MLA) was performed and average of expected heterozygosity was calculated for each loci and haplotype over time.

**Results:**

Three different alleles for CSP, seven for MSP-1 Block 2, one for MSP-1 Block 17, three for AMA-1 and for LSA-1 each and one for TRAP were identified. There were 24 different haplotypes in 125 infections with complete locus typing for each gene.

**Conclusion:**

Characterization of the genetic diversity in *Plasmodium *isolates from the Amazon Region of Peru showed that *P. falciparum *T and B cell epitopes in these antigens have polymorphisms more similar to India than to Africa. These findings are helpful in the formulation of a vaccine considering restricted repertoire populations.

## Background

Vaccine design for *Plasmodium falciparum *is hindered by polymorphisms in certain vaccine candidate loci [1,2]. Highly polymorphic regions have been observed in *P. falciparum *antigenic surface proteins, such as the circumsporozoite protein (CSP), the merozoite surface protein 1 (MSP-1), the apical membrane antigen 1 (AMA-1), the liver stage antigen (LSA-1) and the thrombospondin-related anonymous protein (TRAP) [3].

One of the best characterized and widely accepted by many as a potential vaccine candidate for *P. falciparum *is CSP [4,5]. CSP is a 58-kDa protein and is the major antigen on the surface of malaria sporozoites [6,7]. The CSP protein can be subdivided into two non-repetitive regions (N- and C-termini) and a variable central region consisting of several repeats of four-residue long motifs; both regions exhibit polymorphisms [8-10]. Several T-cell epitopes have been found in the non-repeat regions while immunodominant B-cell epitopes have been identified in the central repeat region [8,11]. RTS, S, AS02, a *P. falciparum *vaccine that consists of the repeat and C-terminal regions of CSP, has successfully completed Phase IIb trials in Mozambique [12,13].

Another antigen that is considered as a vaccine candidate for *P. falciparum *is MSP-1. MSP-1 is a 195-kDa protein that is cleaved into an 83-kDa N-terminal fragment, two central fragments of 30- and 38-kDa and a 42-kDa C-terminal fragment [14,15]. Just before invasion, the 42-kDa is further cleaved into 33- and 19-kDa fragments (MSP-1_33 _and MSP-1_19_). The MSP-1_19 _protein fragment remains anchored to the merozoite surface at the time of erythrocyte invasion and because of its location is a major target of naturally-acquired antimalarial immunity [16]. Within the coding region of the 83-kDa fragment is Block 2, which is a principal target of antibodies associated with clinical immunity in African children [17,18]. In contrast to Block 2, the Block 17 portion of *Pf*msp-1, which encodes the MSP-1_19 _fragment, is conserved with only a few polymorphic sites that produce non-synonymous amino acid changes [16,19].

AMA-1 has also been evaluated for inclusion in a multi-subunit vaccine for both *P. falciparum *and *Plasmodium vivax*. Recombinant AMA-1 induces protective immune responses in mouse and monkey models of malaria [20,21] and both monoclonal and polyclonal antibodies to AMA-1 inhibit merozoite invasion of erythrocytes [22]. As with the other *P. falciparum *vaccine candidate sequences, *Pf*ama-1 is highly polymorphic [23-25] with most of polymorphisms occurring in domain I [22,23,26] making a broadly effective vaccine difficult to create.

The liver stage-specific antigen, LSA-1 is well conserved among *P. falciparum *isolates and is also considered a vaccine candidate. Cytokines, such as interferon gamma, have been implicated in the control of Plasmodium growth and with protection from reinfections with *P. falciparum *[27]. Studies have shown that the N-terminal and *Pf*LSA-1 protein junction (*Pf*LSA-J) regions of *Pf*LSA-1 protein, could induce INF-γ by CD8+ T-cells in adults [28].

Yet another candidate for inclusion in a vaccine for *P. falciparum *is TRAP [29,30]. As with the many vaccine targets discussed above, TRAP protein is highly polymorphic. Studies designed to identify HABPs in TRAP successfully identified 21 loci, three of which contain B epitopes [31], while other studies using INF-gamma ELISPOT identified two CD8+ lymphocyte epitopes [32].

Knowledge of the distribution of polymorphic sites on malaria antigens is necessary to obtain a detailed understanding of their significance for vaccine development. This is the first report of the variants found in this part of the Amazon basin; moreover, this study includes infections occurring early in the Peruvian *P. falciparum *emergence (1998–1999) [33] as well as more recently occurring infections (2003–2006).

## Methods

### Malaria samples

*Plasmodium falciparum *isolates were collected from endemic areas in the Peruvian Amazon Department of Loreto during years 1998 to 2006 using human use approved protocols. Loreto is located in the northeast part of Peru and encompasses 30% of the Peruvian territory. The climate is warm and humid, with the rainy season (December – March) having temperatures reaching 36°C and the driest season (June – July) having temperatures as low as 17°C – 20°C.

There were 139 *P. falciparum *isolates available for this study, all from *P. falciparum*-infected individuals living in or near the city of Iquitos. Twenty-seven of the isolates were collected in years 1998–1999 from patients diagnosed with severe and complicated malaria (courtesy of Dr. Richard Witzig; IRB NMRCD.2005.0004). Twenty-four samples were from patients enrolled in a sulphadoxine-pyrimethamine *in vivo *study conducted in 1999 (WRAIR #719). Thirty-two samples were collected between years 2003 to 2005 (11 from 2003 and 2005 each and 10 from 2004) from a community in the San Juan district, approximately five kilometers south of Iquitos, called Zungarococha from individuals participating in an active malaria detection study, including symptomatic and asymptomatic individuals (IRB PJT.NMRCD.015; IRB UAB) [33]. Also, fifty-six samples from year 2006 came from individuals presenting with malaria-like illness at clinics located in different communities near Iquitos (IRB NMRC.2000.0006). All samples obtained were blood samples confirmed to be positive for *P. falciparum *by microscopy.

### Reference strain

Strain 3D7, which is the current vaccine strain used by the US Department of Defense malaria research programmes [34], was used for comparative genetic analysis with Peruvian sequences.

### Genetic analysis of B- and T-cell epitopes

The genetic diversity of 11 DNA regions encoding mainly T and B cells epitopes in CSP (*Pfcsp*), MSP-1 (*Pfmsp-1 *Block 2 and *Pfmsp-1 *Block 17), AMA-1 (*Pfama*-1), LSA-1 (*Pflsa-1*) and TRAP (*Pfssp-2*) was determined in the isolates. DNA was purified from 200 μL of whole blood using the QIAamp DNA Blood mini kit. PCR fragments were generated using primers and conditions listed in Table 1 in order to amplify DNA regions encoding the selected T or B cell epitopes. PCR amplification was performed in a 30 μL reaction mixture containing 0.2 μM each of forward and reverse primers (for *Pfmsp-1 *0.8 μM of each primer was used), 200 μM each dNTP, 0.6 units of DNA polymerase recombinant, 3 μL of 10× PCR buffer, 1.5 mM of MgCl_2 _and 5 μL of the template extracted from blood.

**Table 1:** List of primers used in this study.

**Gene**	**Regions**	**Epitopes**	**Primers 5' – 3'**	**Ref**
*Pfcsp*	Th2R Th3R	T cell	Th2R_3Rf: ACCATCAAGGTAATGGACAAGG Th2R_3Rr: ACGACATTAAACACACTGGAAC	[8]
*Pfmsp-1*	block 2	T cell	C1_f: AACTAGAAGCTTTAGAAGATGCAG C3_r: ACATATGATTGGTTAAATCAAAGAG	[16]
*Pfmsp-1*	block 17	T cell	M16_f: CCTAATACAATAATATCAAAATTAATTGA C3Fla_r: ATTAAGGTAACATATTTTAACTCCTAC	[16]
*Pfama-1*	Domain I	T cell	amex5_f: GAACCCGCACCACAAGAAC amexe_r: TTGTTTAGGTTGATCCGAAGC	[24]
*Pflsa-1*	N-terminal region	T cell	lsp_f: AAAATCTAACTTGAGAAGTGGTTCT lsp_r: TTCTTGTCTGTTTTCGTCTT	Current manuscript
*Pfssp-2*	Region II	T cell B cell	ssp-2Af: CGTCGTCATAATTGGGTG ssp-2Ar: CCTCTTGGTCTAGGTTGAT	Current manuscript
*Pfssp-2*	Region IV	B cell	ssp-2Cf: GTTATCGGACCCTTTATG ssp-2Cr ATAGGGTGTTGCTGCTCC	Current manuscript

The following PCR conditions were used:

*Pfcsp *genetic variability: 3 min at 94°C, 2 min at 58°C, 2 min at 72°C; 1 min at 94°C, 2 min at 58°C, 1 min at 72°C for 32 cycles and a final extension of 72°C for 10 min; obtaining a PCR product of 318 bp.

*Pfmsp-1 *genetic variability (Blocks 2 and 17): 5 min at 94°C; 30 sec at 91°C, 40 sec at 50°C, 40 sec at 70°C for 40 cycles and a final extension of 70°C for 5 min; obtaining a PCR product of approximately 400 bp.

*Pfama-1 *genetic variability: 2 min at 95°C; 1 min at 95°C, 1 min at 55°C, 2 min at 72°C for 30 cycles and a final extension of 72°C for 2 min; obtaining a PCR product of 803 bp.

*Pflsa-1 *genetic variability: 5 min at 94°C; 30 sec at 91°C, 40 sec at 45°C, 40 sec at 70°C for 40 cycles and a final extension of 70°C for 5 min; obtaining a PCR product of 336 bp.

*Pfssp-2 *genetic variability (Regions II and IV): 5 min at 94°C; 30 sec at 91°C, 40 sec at 50°C, 40 sec at 70°C for 35 cycles; and a final extension of 70°C for 5 min; obtaining PCR products of 1000 bp and 650 bp, respectively.

PCR products were purified using Qiagen PCR spin columns and sequenced using BigDye terminator v3.1 cycle (Applied Biosystem, Foster City, CA) sequencing kit, the primers listed in Table 1 and an ABI 3100 automated sequencer.

### Sequence analysis

Sequences were analysed using the Sequencher version 4.7 software (Gene Codes Corporation, Michigan) and comparative alignments to 3D7 were performed using the MEGA version 3.1 software [35]. Additionally, strains 7G8, D6 and W2 were sequenced and used as regional reference strains. Nucleotide diversity, Pi (π) [36,37], which is the average number of substitutions between any two sequences, and haplotype (gene) diversity values [36] were estimated with the DnaSP 4.0 software [38]. The standard deviation (or standard error) was calculated for both measures. Statistical Analysis Software (SAS Version 9.3, Cary, NC) was used to test multilocus linkage association (MLA) by Fisher Exact Test (FET); two-tailed with alpha = 0.05. The p-value was multiplied by four to correct for conducting pairwise comparisons of four antigens. The antigen diversity was calculated by determining the heterozygosity of alleles detected in each antigen, where heterozygosity (H) = (1 - S*p*^2^), with *p*^2 ^being the squared frequency of each allele variant. Wright's F-statistic (Fst) to evaluate gene flow among the different study years was determined using DnaSP 4.0 software.

## Results

Samples were analysed in three major groups, called *study-years*: 1998–99, 2003–05 and 2006. Amplified products from 139 isolates were obtained for *Pf*csp; 135 for *Pf*msp-1, 128 for *Pf*ama-1, 137 for *Pf*lsa-1 and 131 for *Pfss*p-2. In general, the observed polymorphisms in Peruvian isolates have been previously reported in other populations from South America [9,23]. Nonsynonymous substitutions were found in all regions encoding T and B cell epitopes, except in *Pfss*p-2. From all study years, diversity was lowest in the 1998–99 isolates.

### Pfcsp

DNA sequences of the 3' region spanning the Th2R and Th3R epitopes at the C-terminal portion of CSP were obtained from the 139 isolates. Results from all samples showed four different alleles (Table 2), none of them similar to the 3D7 strain. In comparison to the 3D7 sequence, mutation points were observed at residues 329 (Lys→Gln), 332 (Lys→Glu), 333 (Glu→Gln), 336 (Asn→Lys/Gln), 339 (Gln→Lys/Arg), 341 (Ser→Ala), 342 (Leu→Ile), 367 (Asn→Gly), 369 (Pro→Ser), 372(Glu→Gln), 374 (Asp→Asn) and 376 (Ala→Glu), all located in Th2R and Th3R regions. From the four different alleles found in Peruvian isolates, allele 1 was the prevalent allele in 1998–99 while in 2003–05 and 2006 both alleles 1 and 2 were commonly found (Table 3). The Peruvian *Pf*csp sequences were more closely related to 7G8, HB3 and Ven 765 alleles, which was expected due to the close geographical origin of the isolates [39]. All polymorphisms were non-synonymous, and most mutations were at the first or second position of the codons, corroborating earlier studies [7]. In comparison with 7G8, W2 and HB3 strains, alleles 1 and 2 (Table 2) were exactly the same to 7G8 and HB3 sequences, respectively. It is also interesting to notice that all the isolates in the present study that share identical Th2R sequence also share identical Th3R sequence.

**Table 2:** Alleles found in Peruvian isolates.

**Gene**	**Allele**	**Amino acid sequences**									
*Pfcsp*		**326**	**Th2R**	**342**			**367**	**Th3R**	**378**		
	3D7	P S D K H I **K E **Y L **N **K I **Q **N **S L**					**N **K **P **K D **E **L D Y **A **N D				
	1	. . . . . . **E Q **. . **K **. . **K **. . **I**					. . . . . . . . . **E **. .				
	2	. . . . . . **E Q **. . **K **. . . . . .					**G **. **S **. . . . . . **E **. .				
	3	. . . . . . **E Q **. . **K **. . . . **A **.					. . . . . **Q **. . . **E **. .				
	4	. . . **Q **. . **E K **. . **Q **. . **R **. . .					. . . . . **Q **. **N **. **E **. .				

*Pfmsp-1*		**Block 2**					**Block 17**				
							**1644**	**1691**	**1700**	**1701**	**1716**
	3D7	SAQSGASAQSGASAQSGASAQSGASAQSGASAQSGTSGPSGPSGT					E	T	S	R	L
	1	SAQSGTSGTSGTSGTSGTSGTSGTSGTSGTSGTSGPSGPSGT					Q	K	N	G	f
	2	SGGSVTSGGSGGSGGSGGSGGSVASGGSVASGGSGGSVASGGSVASGG					Q	K	N	G	f
	3	SAQSGASAQSGASAQSGASAQSGTSGPSGPSGT					Q	K	N	G	f
	4	SAQSGTSGTSGPSGTSGPSGTSGPSGTSGPSGTSGPSGT					Q	K	N	G	f
	5	SAQSGASAQSGASAQSGASAQSGASAQSGTSGPSGPSGT					Q	K	N	G	f
	6	SGGSVTSGGSGGSGGSGGSVASGGSVASGGSGGSVASGGSVASGG					Q	K	N	G	f
	7	SAQSGTSGTSGTSGTSGTSGPSGPSGT					Q	K	N	G	f

*Pfama-1*		**259**	**R1**	**271**			**279**	**R2**	**287**		
	3D7	G P R Y C N K D **E **S K R N					A K D **I **S F Q N Y				
	1	. . . . . . . . . . . . .					. . . . . . . . .				
	2	. . . . . . . . **Q **. . . .					. . . **K **. . . . .				
	3	. . . . . . . . . . . . .					. . . **K **. . . . .				

*Pflsa-1*		**84**	**T1**	**107**							
	3D7	L **T **M S N V K N V S Q T **N **F K S L L R N L G V S									
	1	. **S **. . . . . . . . . . . . . . . . . . . . . .									
	2	. **S **. . . . . . . . . . **Y **. . . . . . . . . . .									
	3	. . . . . . . . . . . . . . . . . . . . . . . .									

*Pfssp-2*		**P1**		**P2**		**P3**		**P4**		**P5**	
		**79**	**88**	**201**	**215**	**421**	**435**	**461**	**475**	**504**	**513**
	3D7	NLNDNAIHL		FLVGHPSDGKCNLY		NDKSDRYIPYSPLSP		SEDRETRPHGRNNENRSY		GLALLACAGLAY	
	1	...E....		.............		.............P.		..................		............	

Amino acids sequences for major T and B cell epitopes in vaccine candidate antigens from Peruvian isolat

**Table 3:** Allele frequencies per study-year.

**Protein**	**allele**	**Allele**	**All allele**	**All allele**	**p-value**	**1998–99**	**2003–05**	**2006**
	**code**	**description**	**freq**	**count**	**year-study**	**allele freq**	**allele freq**	**allele freq**
CSP	1	7G8	0.6739	93		0.8823	0.5625	0.5357
CSP	2	HB3	0.3043	42		0.0784	0.4375	0.4286
CSP	3	Ven765	0.0217	3		0.0196	0	0.0357
CSP	4	peru1	0.0072	1		0.0196	0	0
p-value testing year-study difference; main observation:					**0.0001 ;**	**CSP allele 1 decreasing**		

MSP-1-B2	1	K1-126	0.4962	67		0.4898	0.5625	0.463
MSP-1-B2	2	MAD20-144	0.363	49		0.4082	0.25	0.3889
MSP-1-B2	3	K1-99	0.0593	8		0	0.1563	0.0556
MSP-1-B2	4	K1-117	0.0296	4		0	0	0.0741
MSP-1-B2	5	K1-117	0.037	5		0.0816	0	0.0185
MSP-1-B2	6	MAD20-135	0.0074	1		0	0.0313	0
MSP-1-B2	7	K1-81	0.0074	1		0.0204	0	0
K1 total allele count: 85; MAD20 total allele count: 50								
p-value testing year-study difference ; main observation:					**0.0167 ;**	**MSP-1-B2 allele 4 increasing**		

AMA-1	1	3D7	0.8438	108		0.8542	0.8214	0.8462
AMA-1	2	peru1	0.125	16		0.1458	0.1786	0.0769
AMA-1	3	7G8	0.0313	4		0	0	0.0769
p-value testing year-study difference ; main observation:					**0.1419;**	**AMA-1 allele 3 increasing**		

LSA-1	1	SN	0.7391	102		0.5098	0.7742	0.9286
LSA-1	2	TN	0.2464	34		0.4902	0.2258	0.0357
LSA-1	3	SY	0.0145	2		0	0	0.0357
p-value testing year-study difference ; main observation:					**<0.0001;**	**LSA-1 allele 1 increasing**		

According to DNA polymorphism analysis per study-years, haplotype diversity values have increased since 1998–99 (0.21882) until 2006 (0.53766); also, the nucleotide diversity value for 2006 samples (0.01252) showed an increment of almost 2.3 times more comparing to the value obtained for samples from years 1998–99 (0.00539).

### Pfmsp-1

The genetic variability in Blocks 2 and 17 of *Pfmsp-1 *was determined and compared to the vaccine strain 3D7. From the 135 samples analysed, 85 samples had the *Pfmsp-1 *Block 2 (*Pfmsp-1-B2*) K1 type and 50 had the *Pfmsp-1-B2 *MAD-20 type. Seven different *Pfmsp-1-B2 *alleles were found, five from the K1 type and 2 from the MAD-20 type (Table 2). *Pfmsp-1-B2 *allele frequencies differed by study-year. Considering protein (MSP-1B2) allele frequencies, study-year 1998–99 only had two MSP1B2 alleles at a frequency of >10%, while study-year 2003–05 had three MSP1B2 alleles at a frequency of >10% (Table 3).

Allele 1 (a K1 type) and Allele 2 (a MAD-20 type) were the most frequently found in all Peruvian samples. K1 type alleles commenced with the hexapeptide SAQSGT or SAQSGA and ended with SGPSGT. Most diversity was due to duplications or deletions of the repeat motifs SAQ, SGT and SGP. The MAD-20 type allele started with SGGSVT and ended with SVASGG and diversity was due to repetitions of SGG and SVA. Synonymous substitutions were observed for Alanine (GCA/GCT) in K1 isolates and for Glycine (GGC/GGT) in MAD-20 isolates. Also, Block 2 PCR products of the same size did not necessarily correspond to the same allele family since there were two K1 alleles that were the same base-pair length, but had different sequence (Table 3).

Nucleotide sequences for the C-terminal 19-kDa region of MSP-1 (Block 17) were also determined. Substitutions at amino acid positions 1644 (Glu→Gln), 1691 (Thr→Lys), 1700 (Ser→Asn), 1701(Arg→Gly) and 1716 (Leu→Phe) have been previously reported [16,40] but all isolates from Peru only contained amino acids Gln, Lys, Asn, Gly and Phe in these positions.

### Pfama-1

The alignment of *Pf*AMA-1 domain I included 128 isolates. Regions 259–271 (R1) and 279–287 (R2) are naturally immunogenic T and B cell epitopes and non-synonymous substitutions were found within these resgions in positions 267 (Glu→Gln) and 282 (Ile→Lys) (Table 2). Overall *Pfama*-1 Domain I Pervian nucleotide sequences, 24 variable sites were found, 23 of them corresponding to nonsynonymous substitutions and only one corresponding to a synonymous substitution. Three different alleles were found in Peru; from which allele 1 had identical amino acid sequence to the vaccine strain 3D7 with only one nucleotide different (AAA/AAG) in codon 292 (Lys). This was the allele most commonly found, followed by allele 2, and then followed by allele 3, which was only detected in study-year 2006 (Table 3).

### Pflsa-1

Sequences of the N-terminal region of *Pflsa-1*, designated T1, were aligned from 137 isolates with the 3D7 sequence. The N-terminal region of LSA-1 has been shown to induce interferon gamma production in peripheral blood mononuclear cells (PBMC). The alignment showed two non-synonymous substitutions in aa 85 (Thr→Ser) and aa 96 (Asn→Tyr) and that the most abundant allele for T1 was allele 1. Alleles 2 and 3 were less frequent (Table 3), the latter having the same nucleotide sequence as 3D7 (Table 2).

### Pfssp-2

The antigenic variability of TRAP, encoded by *Pfssp-2*, was assessed at previous discovered CD8+ T-lymphocyte TRAP epitopes located at amino acids 78–88 (P1) and 504–513 (P5) as well as at B cell epitopes located at amino acids 201–215 (P2), 421–435 (P3) and 461–475 (P4) [31] (Table 2). The alignment of 131 sequences revealed that epitopes corresponding to P1, P2, P3, P4 and P5 are highly conserved in Peruvian isolates. In comparison to the 3D7 sequence, two substitutions were found at positions 82 (Asp→Glu) and 434 (Ser→Pro), and all Peruvian isolates had the same sequence.

### Multilocus analysis

For each sample, the combination of alleles detected in the MSP-1B2, CSP, AMA-1, and LSA-1 (multilocus haplotypes) were determined. The order, listed above, was based upon pairwise multilocus association (MLA) analysis demonstrating the most consistent linkage between MSP-1B2 and the other antigens. The result is a four digit code where each digit is the allele detected for the respective loci: twenty-four different multilocus haplotypes were detected (Table 4). Considering all study-years (1998–99, 2003–05, and 2006 samples all together), MSP-1B2 alleles were associated with each of the other loci (p ≤ 0.0001, Fisher Exact Test [FET]) and CSP alleles were associated with MSP-1B2 and AMA-1 (p ≤ 0.0001, FET). CSP was also associated with LSA-1 (p = 0.0003, FET); however, the linkage was less specific: 47.5% were allele 1 and 1, but 19.0% were allele 1 with 2 and 25.6 were allele 2 with 1. AMA-1 was only associated with LSA-1, and this was marginal (p = 0.0401). The only linkage that was observed in each of the year-study samples was MSP-1B2 with AMA-1 (p < 0.0142, FET), with the allele 1 and allele 1 (and NOT allele 1 and NOT allele 1) linkage being observed each year-study. The linkage between CSP and AMA-1 and the linkage between CSP and MSP-1B2 was observed in the 1998–99 study-year and the 2006 study-year. The MSP-1B2 and LSA-1 allele linkage (allele 1 with 1 and allele 2 with 2) was only observed in 1998–99. The only MLA observed in the 2003–05 samples (active case detection) was between MSP-1B2 and AMA-1 (p = 0.0005, FET), with MSP-1B2 allele 3 and AMA-1 allele 2, being detected together 14.3% of the time.

**Table 4:** Haplotypes defined by allelic variants (1–7) of four antigens MSP-1-B2:CSP:AMA-1:LSA-1 (respectively), comparing the 1998–99, 2003–05 and 2006 study-years.

**Haplotypes** MSP-1:CSP:AMA-1:LSA-1	**All**		**1998–99**		**2003–05**		**2006**	
**1111**	**46**	36.2%	19	40.4%	6	21.4%	21	42.0%
**1112**	**5**	3.9%	4	8.5%	1	3.6%	0	0.0%
**1131**	**1**	0.8%	0	0.0%	0	0.0%	1	2.0%
**1211**	**6**	4.7%	0	0.0%	5	17.9%	1	2.0%
**1212**	**2**	1.6%	0	0.0%	2	7.1%	0	0.0%
**2111**	**3**	2.4%	0	0.0%	1	3.6%	2	4.0%
**2112**	**16**	12.6%	14	29.8%	1	3.6%	1	2.0%
**2121**	**1**	0.8%	1	2.1%	0	0.0%	0	0.0%
**2122**	**3**	2.4%	2	4.3%	1	3.6%	0	0.0%
**2211**	**20**	15.7%	0	0.0%	4	14.3%	16	32.0%
**2212**	**2**	1.6%	1	2.1%	1	3.6%	0	0.0%
**2422**	**1**	0.8%	1	2.1%	0	0.0%	0	0.0%
**3121**	**6**	4.7%	0	0.0%	4	14.3%	2	4.0%
**3211**	**1**	0.8%	0	0.0%	0	0.0%	1	2.0%
**3212**	**1**	0.8%	0	0.0%	1	3.6%	0	0.0%
**4212**	**1**	0.8%	0	0.0%	0	0.0%	1	2.0%
**4231**	**3**	2.4%	0	0.0%	0	0.0%	3	6.0%
**5121**	**1**	0.8%	1	2.1%	0	0.0%	0	0.0%
**5212**	**1**	0.8%	1	2.1%	0	0.0%	0	0.0%
**5323**	**1**	0.8%	0	0.0%	0	0.0%	1	2.0%
**5211**	**1**	0.8%	1	2.1%	0	0.0%	0	0.0%
**5221**	**1**	0.8%	1	2.1%	0	0.0%	0	0.0%
**6111**	**1**	0.8%	0	0.0%	1	3.6%	0	0.0%
**7111**	**1**	0.8%	1	2.1%	0	0.0%	0	0.0%
**N =**	**125**		**47**		**28**		**50**	

**Count of haplotypes =**		24		12		12		11
**Haplotype diversity (Hd) =**		**0.7932**		**0.6910**		**0.8466**		**0.7543**
**Hd standard deviation =**		0.028		0.055		0.033		0.045

Multilocus nucleotide genetic diversity was determined and compared for each study-year sampling (1998–99, 2003–05, and 2006). Combining all loci together, the nucleotide diversity was similar in all study-years (ranging from 0.2725 to 0.2794); however, the samples collected in study-year 2003–05 (samples collected in active case detection) had higher amino acid haplotype diversity (0.8466 +/-SD 0.033) in comparison to the 1998–99 and 2006 study-years (0.6910 +/- SD 0.055 and 0.7543 +/- SD 0.045, respectively). Likewise, comparing the average allelic heterozygosity for each antigen, the average across all antigens was highest in the 2003–05 samples (H = 0.4474) versus 1998–99 (H = 0.4366) and 2006 (H = 0.3975).

The genetic distance, or gene flow, among the 1998–1999, 2003–2005 and 2006 study-years was determined using Wright's F-statistic, Fst. The Fst for each pairwise comparison was low, ranging from -0.0168 to -0.0050. Therefore, there was no evidence of genetic isolation by year-study. The 2003–2005 and 2006 study-years were the most genetically similar (Fst = -0.0168).

## Discussion

Investigating the extent of genetic variation can assist in the laborious process required in selecting antigens for further vaccine development. Several *P. falciparum *and *P. vivax *vaccine candidate antigens are highly polymorphic which could pose a serious problem in the formulation of vaccines from a single, well-characterized strain. Previous studies have addressed the diversity and maintenance of several malarial vaccine candidates in Africa [6]. An understanding of genetic variability in T and B cell epitopes of vaccine candidate antigens in *P. falciparum *Peruvian isolates from the Amazon basin is presented in this study.

According to the *Pf*csp nucleotide and haplotype diversity values, Th2R and Th3R regions have higher values than those obtained for other antigens in this Peru study; nevertheless, *Pf*csp nucleotide and haplotype diversity values in Peru for Th2R and Th3R regions are much lower than those obtained in Kenya (**π **= 0.09772 and 0.07442), Gambia (**π **= 0.08789 and 0.06454) and Venezuela (π = 0.08466 and 0.08025). However, Peruvian isolates exhibit similar Th2R nucleotide diversity values to samples from Brazil, Vietnam and India [7,9]. Allele 1 has been previously reported in Iran [41], Brazil [42] and Gambia [43]; allele 2 in Thailand [44], Iran [41] and Myanmar (de Stricker et al., unpublished); and allele 3 in Venezuela [9].

In addition, haplotype diversity for the entire 3' end of CSP was influenced by both Th2R and Th3R regions exclusively, since all 7 variable sites were contained in these regions and all of the polymorphims produced nonsynonymous substitutions. Linkage and recombination events between the Th2R and Th3R regions have been described in previous studies [9]. In the present study, haplotype linkage between *Pf*csp Th2R and Th3R polymorphisms was also observed. Nucleotide and haplotype diversity values for the entire 3' end of CSP of all samples were 0.01102 and 0.46387, respectively. These values show that Peruvian isolates are less diverse than African and more similar to Indian isolates [9].

CSP is one of the most widely characterized malaria vaccine candidate antigens, and the only one whose components have gone so far as completion of a successful phase II b clinical trial [12,45], the generation of relevant genetic, epidemiologic and immunologic data for the CSP gene is important, particularly for regions of low malaria endemicity. It is apparent that diversity in *Pf*csp is regionally restricted, and that Peru has low genetic polymorphism with one predominant allele and variants in a small number, as in India [10], Vietnam [7] and Thailand [5]. This suggests that since polymorphisms are restricted and can be grouped, allelic variants can be included in a polyvalent vaccine that could be widely effective.

On the other hand, diversity in *Pfmsp*-1 Block 2 was lower than reported in *P. falciparum *isolates studied in other geographic regions [16]. Neither RO33 nor MR Block 2 alleles were found; although these Block 2 alleles have been reported in other studies, including Venezuela [15,46]. The number of alleles detected using this direct sequencing method is lower than those detected using PCR-based genotyping methods of samples from other populations such as Kenya [15] with 20 K1 and 15 MAD-20 alleles detected by nested PCR from 362 samples. However, similar results to the Peruvian samples were found in 104 isolates from Brazil with eight alleles verified by Southern blot and SSCP-PCR [47]. It is important to remark that the direct sequencing used in this Peruvian study only detected one *Pfmsp-1 *Block 2 per infection (sample) and, therefore, could underestimate the genetic diversity by only detecting the more high-density/dominant allele in each infection. Results indicated that 63% of the isolates in this study where K1 alleles. Although the 3D7 vaccine strain is in the K1 allele family, none of the Peruvian isolates had the same K1 allele (sequence) as the vaccine strain 3D7.

Strikingly, nucleotide sequences for the C-terminal 19-kDa region of MSP-1 (Block 17) showed no diversity at positions 1644 (Gln), 1691 (Lys), 1700 (Asn), 1701 (Gly) and 1716 (Phe), while polymorphisms in these positions have been reported in Brazil, Vietnam and Kenya [48,49]. The 3D7 vaccine strain has the MSP-1 19 kDa haplotype ETSRL, suggesting that if immunity conferred by monovalent vaccines is allele specific, it would have low overall efficacy in populations where the target allele is in the minority [50].

Results also showed two non-synonymous substitutions in R1 and R2 regions of *Pfama*-*1*. The extensive number of non-synonymous polymorphisms outside R1 and R2 described in this study revealed that this gene could undergo intense selective pressure in these other regions of AMA-1 upon administration of a full-length peptide vaccine having a single allele type. The overall genetic diversity in *Pfama-1 *domain I (π = 0.00697) is lower than in the Kenyan and Southeast Asian isolates (π values of 0.01361 and 0.01196, respectively) [23]. From the three different *Pfama-1 *alleles found, allele 3 was only present in samples from 2006 and could have arisen by a new introduction of this R1-R2 haplotype from another geographic region or by recombination between alleles 1 and 2 which existed in prior study-years.

Two non-synonymous and no synonymous substitutions were found in LSA-1 T1 in Peruvian isolates. In comparison, within Brazilian, Papua New Guinean and Kenyan isolates two synonymous substitutions (positions 87 and 104) and three different non-synonymous substitutions (positions 92, 95 and 104) have been reported [51]. These data suggest that T cell epitopes of *P. falciparum *LSA-1 are highly conserved in field isolates from geographically diverse regions with varying transmission patterns, since non much allelic diversity is observed. However, even in this low and recent transmission region of Peru, we detected two non-synonymous substitutions.

*Pf*ssp-2 HABPs sequences were highly conserved among these Peruvian isolates. Part of the peptide 2 sequence has been reported to be a B cell epitope, recognized by sera from malaria immune humans living in endemic areas [52]. Peptides 3 and 4 are located within a region characterized by the presence of a great number of Asn, Lys and Pro residues. This long stretch of amino acids suggests configuration binding sites, which could be altered with just minimal changes in amino acid sequence [31]. For instance, results showed Pro in site 434 instead of Ser as in the 3D7 sequence; also, P3 showed a characteristic sequence in which amino acids Asp and Pro are present each forth residue and that may allow binding in different registers [28]. P1 and P5 have been identified as CD8+ T-lymphocyte epitopes and also showed to be highly conserved, with just one variation in aa 82 (Glu/Asp) compared to 3D7 strain.

Most of the genetic diversity detected in Peruvian isolates was attributable to non-synonomous, amino acid changes. Over time, comparing the allele frequencies in the 1998–1999, 2003–2005 and 2006 study-years, there were changes in allele frequencies with new alleles being detected. The 2006 samples had the highest allele counts. Although multilocus associations were detected, these associations were most evident in the year 1998–1999 samples. Interestingly, the 2003–2005 study-year samples had the least multilocus associations (only MSP-1B2 and AMA-1 polymorphisms were determined to be associated) and had the greatest overall allelic diversity (H = 0.8466). The diversification observed over time might be attributed to natural selection from a specific immune response. In particularly, regarding the 2003–2005 samples, these samples were collected in active case detection in a population where the frequency of asymptomatic infections suggests immune pressure [37].

Several studies have demonstrated the importance of T and B cell mediated immunity to malaria and how nonsynonymous substitutions may affect the conformation of epitopes and in consequence the immunological response [53]. Nevertheless, more studies need to be done in order to understand the immunological implications of amino acid changes in these malaria vaccine candidate antigens. Studying different genes and their alleles help us to understand if they interact to influence in malaria infection and if minimal changes in their sequence could render a vaccine ineffective. An alternative to improve the effectiveness of a vaccine would be to create a construct using the most common regional specific alleles considering the genetic diversity found in the area. By providing information about the prevalence and dynamics of vaccine candidate antigens polymorphisms, an accurate construct could be built.

## Conclusion

Peruvian isolates are less polymorphic than African and more like Indian populations. Although, conserved epitopes were found in Peru, the observation of uneven geographic distribution of polymorphisms and the high number of alleles distributed worldwide, especially for CSP and MSP-1 Block 2 may have an adverse impact on the effectiveness of vaccines. The number of allelic variants increased over time in this study, suggesting that even in geographic regions with low transmission, vaccine strategy development should include conduction of diversity studies. The uneven geographic distribution of alleles may jeopardize the formulation and use of vaccines directed on a specific variable loci since local variants may not be considered in the vaccine design.

## Abbreviations

CSP: Plasmodium falciparum circumsporozoite protein; MSP-1: Plasmodium falciparum merozoite surface protein-1; AMA-1: Plasmodium falciparum apical membrane antigen-1; TRAP: Plasmodium falciparum thrombospondin related anonymous protein; LSA-1: Plasmodium falciparum liver stage antigen-1; HABP: high affinity binding peptide; *Pfcsp*: gene encoding CSP; *Pfmsp-1*: gene encoding MSP-1; *Pfama-1*: gene encoding AMA-1; *Pfspp-2*: gene encoding TRAP; FET: Fisher Exact Test; SD: standard deviation

## Glossary

Interferon-gamma (IFN-γ): a dimerized soluble cytokine that is the only member of the type II class of interferons.

T cell epitopes: antigenic determinants recognized and bound by the T-cell receptor.

B-cell epitopes: antigenic determinants recognized and bound by the B-cell receptor.

## Authors' contributions

SMC conducted the molecular genetic studies, analysed the data and wrote the first draft of the manuscript, OB provided isolates, participated in the genetic diversity, linkage, and study-year comparisons and assisted in writing and editing the manuscript, AAE participated in the DNA polymorphisms analysis and assisted in writing and editing the manuscript, CML participated in the coordination of the laboratory component of the study and assisted in editing the manuscript, DJB designed the project, supervised and directed the research team and contributed to the writing and editing of the manuscript. All authors read and approved the final manuscript.
